# Conceptualising integration: a framework for empirical research, taking marriage migration as a case study

**DOI:** 10.1186/s40878-016-0035-x

**Published:** 2016-10-06

**Authors:** Sarah Spencer, Katharine Charsley

**Affiliations:** 1 Centre on Migration, Policy and Society, University of Oxford, 58 Banbury Road, Oxford, OX26QS UK; 2 School for Sociology, Politics and International Studies, University of Bristol, 11 Priory Road, Bristol, BS8 1TU UK

**Keywords:** Integration, Marriage, Migrants, Family, Gender, Minorities, Transnational, Research method, Policy

## Abstract

Enquiry into the factors which impact on ‘integration’ requires clarity on the nature of the integration processes in which individuals are engaged, the intersection of those processes and the factors that may affect their operation over time. Elaborating on debates among European scholars which conceptualise integration as a series of multi-directional, inter-active processes in related but separate domains, we use the term ‘effectors’ to explore five sets of factors which have been shown to facilitate or impede those processes, setting out a framework capable of empirical and comparative application. We demonstrate the utility of this model in a case study funded by the Economic and Social Research Council (2013–2015) exploring the impact of transnational marriages in the UK, illustrating the conceptual and empirical value of the model when investigating the complexity of the factors involved in shaping the outcomes of integration processes. The model is illustrated in diagrammatic form. The case study in turn informs the model, highlighting the relevance of family and life-course events within an understanding of the full range of factors impacting on the integration processes in which individuals are engaged.

## Introduction

Enquiry into the factors which impact on ‘integration’ requires clarity on the nature of the integration processes in which individuals are engaged and the factors that may affect their operation over time. This paper, in which those processors and factors are explored, is informed by an ESRC funded study, *Marriage Migration and Integration*, which the authors have, with others (Marta Bolognani, Hiranthi Jayaweera and Evelyn Ersanilli)*,* conducted. This two year collaborative project between the Universities of (Bristol and Oxford) explored the relationship between marriage-related migration and integration processes, drawing on quantitative data sets and qualitative research with the two largest ethnic groups involved in the UK, Indian Sikhs and Pakistani Muslims^1^. We found it necessary to clarify our understanding of integration and what is known from earlier empirical findings of the factors which may impact upon it, in order to set out a framework capable of empirical and comparative application. Only in this way could we identify the potential relevance of marriage with a partner from abroad among other factors. Understanding the full range of potential factors ensures, moreover, that we do not fall into the trap of essentializing individuals and their culture at the expense of the broader socio-economic and structural forces at play (Martiniello, 2013, p. 11).

Spousal migration is a particularly suitable case study in relation to integration processes. Not only is it a significant source of cross-border mobility but it highlights several factors which may impact on integration such as the family, life course and gender. Academic and policy discourses are replete, moreover, with claims about the impact of transnational marriage on integration but differ, explicitly or implicitly, in the meaning they attach to ‘integration’ and lack clarity on the range of factors which impact upon it, within which marriage migration may play a part.

### Marriage migration as a case study

Spouses have long been a significant category of migration in Western Europe. Across the European Union (EU), 28 % of residence permits for non-EU nationals in 2013 were issued for family reasons, including spouses, compared to 23 % for work and 20 % for education (European Commission, 2015). At EU level there is recognition of the importance of family reunion for integration and for the stability of society, the preamble to the EU Directive on Family Reunification^2^ stating:

Family reunification is a necessary way of making family life possible. It helps to create sociocultural stability facilitating the integration of third country nationals in the Member State, which also serves to promote economic and social cohesion, a fundamental Community objective stated in the Treaty.

At Member State level, however, concern is regularly expressed about the cultural and economic implications of transnational marriages – particularly those between members of ethnic minorities and partners from (ancestral) countries of origin (Beck-Gernsheim, 2007; Çelikaksoy, Nielsen, & Verner, 2006; Joppke, 2009; Migration Watch, 2004, 2005; Timmerman, 2006). Such concern has led Denmark since 2000 to impose ever tighter restrictions on spousal immigration (Jørgensen, 2012) and likewise more recently Germany, the Netherlands and the UK. Whilst ethnic intermarriage has been seen as a significant indicator of integration (Beck-Gernsheim, 2007, p. 272; Schinkel, 2011, p. 101; Song, 2009), the arrival of a first generation in every generation is seen as thwarting the process through which migrants and their descendants would otherwise have been incorporated (Charsley, Bolognani, & Spencer, 2014; Crul & Vermeulen, 2003). These discourses also tend to be highly gendered, with a focus on migrant women (from Muslim countries in particular) who are often portrayed as ‘bearers of a backwards and illiberal culture’ and lacking in education and skills, with consequences both for their own integration and that of their future children (Kofman, Saharso, & Vacchelli, 2015, p. 85).

The literature containing such assertions and the partial and varying evidence base on which it draws has been well critiqued elsewhere (Bonjour & Kraler, 2015; Charsley et al., 2014). Here, however, we wish to highlight the varying conceptualisations and operationalisations of ‘integration’ on which they are based. Some writing in this area is based on ‘common sense’ assumptions about the effects of marriage migration (e.g., Collier, 2013; Goodhart, 2013; Joppke, 2007, 2009) assumptions often divorced from insights in the broader social science literature – as for example when strengthening of intra-ethnic ‘bonding’ social capital, or transnational activities, are assumed to have uniformly negative implications for broader processes of integration. Whether the subject of the discussion is the integration of individuals or ethnic groups is another point of variation. Studies may focus on particular aspects of integration – for example gender norms and employment (Dale & Ahmed, 2011; Timmerman, 2006) at the expense of integration processes in other domains, while drawing conclusions that refer to ‘integration’ per se.

Many studies which are sources of evidence on the relationship between marriage migration and integration do not take ‘integration’ as their analytical frame, instead being concerned, for example, with partner choice among ethnic minorities (Carol, Ersanilli, & Wagner, 2014; Çelikaksoy et al., 2006; Gonzalez-Ferrer, 2006). Notwithstanding the contributions that these studies make to knowledge of issues associated with transnational marriage, and the evidence they provide for discussions around marriage migration and integration, the advantage of an ‘integration’ perspective is that it draws our attention to the value of contextualising such data in a broader appreciation of the social and structural complexities in which such these processes unfold, and in which the meanings of particular outcomes must be understood.

### Towards a concept of integration

The terminology of ‘integration’ is, however, by no means unproblematic, bearing connotations on the nature of the process involved and associated policy aspirations that have been challenged by scholars and policy advocates alike. Critiques of the normative basis of integration discourses, and their (somewhat ironic) exclusionary potential are by now well developed (e.g., Rytter, 2010; Schinkel, 2011). It has been argued that the term integration implies the insertion of a group or individual into an existing entity (a society, bounded by a nation state, Favell, 2010, p. 372), and a one way process that neither fits reality nor is a model to which policy should aspire. The terms ‘inclusion’ and ‘incorporation’ have been used by scholars and policy makers as alternatives (Hochschild, Chattopadhyay, Gay, & Jones-Correa, 2013; Martiniello & Rath, 2010). For some, inclusion is preferred because the term is used to address the social exclusion of other marginalised groups, thus bringing migrants into the mainstream (Rudiger & Spencer, 2003, p. 5), rather than because linguistically it has more appropriate connotations. The Cambridge online dictionary definition of inclusion, for instance, is ‘to contain something as part of something else, or to make something part of something else’; while the definition of incorporation is ‘to include something as part of something larger’. Neither capture the nature of the processes which, we shall suggest below, are those that empirical research has demonstrated are at play^3^.

It is interesting to note that this debate on terminology has been mirrored in the field of special education in Europe. ‘Inclusion’ has replaced ‘integration’ in order to convey a ‘broader vision’ embracing the whole school in preference to a focus solely on the children with special needs (Vislie, 2003): a debate in which the actual meaning of ‘inclusion’, as in our field, appears less significant than the need to show that the debate has moved on. We do not conclude that ‘integration’ is the ideal term. Rather, in the absence of a more appropriate alternative, and bearing in mind the need for a mutual vocabulary with which to engage critically with existing academic and policy discourses, we seek to develop a more systematic and nuanced analysis of integration processes that avoids the pitfalls which integration’s critics have rightly identified.

In contrast to early academic analysis of the experiences of migrant newcomers which identified a largely one-way trajectory of cultural assimilation of the minority into a majority ‘host’ society (Alba & Nee, 1997), European and North American multi-disciplinary analysis has since identified more complex processes at play (Ager & Strang, 2008; Entzinger, 2000; Garcés-Mascareñas & Penninx, 2015; Heckmann & Schnapper, 2003; Joppke, 2013; Martiniello & Rath, 2010). Integration processes – of participation, personal and social change – were found to be ‘two-way’: engaging not only the newcomer or member of a marginalised group but also other residents - an interaction which is fundamental to the outcome. While many analyses in practice still focus largely on the newcomer, it is not only they who are engaged: in the labour market, for instance, where employers also necessarily play a role. Likewise, it is not only the newcomer whose values may change but those of other residents with whom they interact:

Thus integration research must not only be on immigrants, but also on natives and the openness of their institutions. Barriers to integration, be it individual or structural forms of discrimination are thus an integral part of integration research’ (Heckmann, 2006, p. 14).

Integration processes were also recognised to be two-way in the sense that they do not proceed in only one direction, from ‘not integrated’ to ‘integrated’ (Phillimore, 2012). ‘Progress’ (to use a normative term) may reverse: through redundancy from work, perhaps, or disillusionment with and disengagement from the democratic process. Integration may also take new forms or directions: disability, for instance, may lead to withdrawal from the labour market but to increased engagement with civil society. There is thus no integration ‘end-state’, no ‘integrated society’ but rather an ever evolving process. Outcomes measured at any one time are a snap-shot, not a permanent feature.

**Fig. 1:**
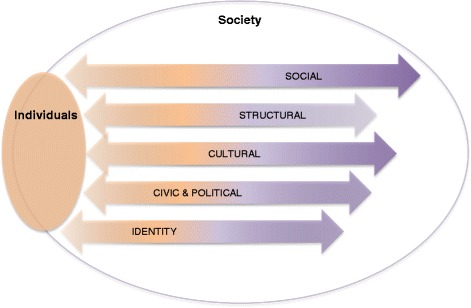
Integration as two way processes across domains

Integration is not a single process but takes place across a series of domains (Fig.1). While categorised slightly differently by scholars, these are in essence *structural* (as in participation in the labour and housing market, education and training); *social* (social interaction, relationships, marriage); *cultural* (changing values, attitudes, behaviour and lifestyle); *civic and political participation* (in community life and the democratic process) and in relation to *identity* (that is, the processes through which individuals develop at some level a shared identity and sense of belonging with the place, nation, communities and people among whom they live (Ager & Strang, 2008; Entzinger, 2000; Heckmann & Schnapper, 2003; Spencer, 2011). This categorisation is, to an extent, a heuristic device: the separation between domains is less evident in some social contexts than others (employment in a family run business illustrates the blurring of boundaries) but helpful in ensuring we recognise some implications, below.

We illustrate this understanding of integration with a series of diagrams of which the last, Fig. 3, presents the heuristic model we propose.

### Differing experiences between and across domains

We know that the integration process in one domain may develop differently to that in another. Individuals may be employed but socially isolated outside the workplace. They may be fully participating in the structural, social and civic and political domains but develop no sense of shared identity and belonging. Shifts in identity may happen more slowly than integration processes in other domains (Heckmann, 2006, p. 17). Likewise in the political domain, participation in the democratic process is largely reserved for nationals of that country, and may be considered less important to migrants and policy makers than economic and social domains (Joppke, 2013, p. 65), though that would not be held true for participation in civil society (Ambrosini & Abbatecola, 2004; Penninx & Martiniello, 2004; Zetter, Griffiths, Sigona, & Hauser, 2003).

Experience in one domain may affect those in another: positively (as where welcoming social contact fosters a sense of belonging); or negatively - if anti-social working hours inhibit opportunities for social engagement or to attend language classes (Spencer, Ruhs, Anderson, & Rogaly, 2007) or poor housing affects health or ability to feel ‘at home’ (Ager & Strang, 2008, pp. 178-180). Labour market participation in particular has been found to have a significant effect on migrants’ experiences in other domains including, in the cultural domain, on attitudes of other residents towards them (Özdemir, Veysel, Uslucan, H.H., Uslucan, S., & Erdem, 2004). We also know that there can be positive trade-offs between domains: remaining within a minority ethnic culture can in some circumstances enhance education and employment prospects for migrants and the second generation (Maxwell, 2012; Portes, Fernandez-Kelly, & Haller, 2005, p. 1013) while for refugees the lack of such contact can have a negative impact on mental health (Ager & Strang, 2008, p. 178). Understanding the impact of what happens in one domain on experiences in another is crucial to a comprehensive analysis of the integration processes underway. As Ager and Strang highlight, understanding this interdependence of domains is also important for policy and practice in the field (Ager & Strang, 2008, p. 185).

The majority society is not homogenous but itself differentiated by many characteristics including class, income, region and age (reflected in the mosaic background of society in our model). It follows that integration processes in some or all domains may relate to one sub-section of society rather than a broader engagement; that is, ‘segmented assimilation’ (Zhou, 1997). The individual may themselves be a member of a tight or loose-knit family or community network which significantly affects their experiences, through cultural expectations or more practically through the benefit of networks that facilitate engagement (Rath & Kloosterman, 2003). Equally, however, family and community may not feature large in the individuals’ experiences.

### Spacial and temporal dimensions

**Fig. 2:**
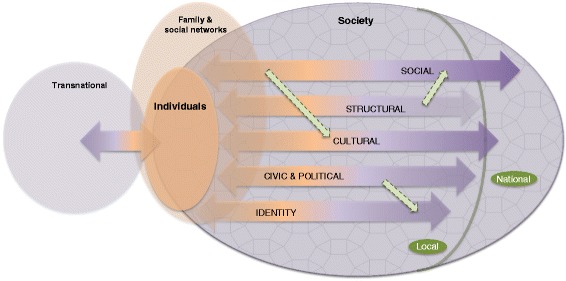
Integration as inter-related, multidirectional, processes across domains

There is also a spacial dimension, most processes taking place at the local level (Caponio & Borkert, 2010; Schmidtke, 2014), reflected in the local/national distinction in Fig. 2. Place matters, bringing differing opportunities and constraints, so that integration ‘can evolve in distinctive ways in different places’ (Platts-Fowler & Robinson, 2015, p. 476). There is also a national dimension (as in national identity) and transnational, as in ongoing social and economic connections and transnational identification – hence integration is not in fact ‘two-way’ but multi-directional (Snel, Engbersen, & Leerkes, 2006).

A focus on temporality brings further insights. Integration has conventionally been associated in scholarship (as in policy) with settlement, relevant to those newcomers intending and entitled to remain in the long term (Heckmann, 2006, p. 13). With the more fluid migration patterns of today, and with the concept of integration processes we outline, it is easier to see that integration processes begin with the first moment of engagement: for the newcomer on the day of arrival (if not before, through transnational contact with family and friends, anticipatory socialisation and, in practical terms, pre-entry integration programmes). It would thus be a misnomer to think of integration as relevant only for those staying long term or indeed relevant only for those with a legal right to stay. That those notions have crept into academic literature (which only with rare exceptions, for instance, relates integration to irregular migrants (Cook, 2013)) demonstrates the way in which policy discourse has influenced academic discourse on integration and the difficulty there can be in separating the normative *ought* from the empirical *is*. This is not to suggest that any analysis of integration processes can be entirely without normative assumptions but it is to contend that it is possible to explore processes that are taking place without making assumptions on whether a ‘right’ outcome has emerged.

### Effectors: impacting on integration processes

Empirically it has been shown that many factors impact on integration processes across the domains, including ‘facilitators’ like language and cultural knowledge (Ager & Strang, 2008), but also barriers that can impede these process such as discrimination, non-recognition of qualifications and restrictions on participation related to immigration status (Heckmann, 2006, p. 16). In our conceptual framing of integration we give prominence to these factors. As they may impact positively, facilitating engagement, or negatively, forestalling it, we employ the term used for that phenomenon in physical science (notably bio-chemistry): ‘effectors’.

**Fig. 3:**
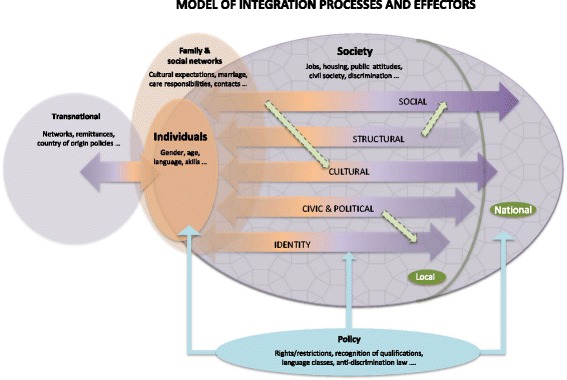
A conceptual model of integration processes and effectors

Figure 3, a conceptual map rather than a depiction of empirical processes, highlights the importance of identifying the full range of effectors potentially at play.

We can identify five sets of effectors from the literature, relating to:

- individuals
- families and social networks
- opportunity structures in society
- policy interventions and
- transnational effectors, which may equally impact through families or policy, for instance, but important to identify in their own right.

First, there are effectors that relate to *individuals,* the forms of human capital that they bring to the table: notably education, skills and language capacity; cultural attitudes and motivation and, for instance, their knowledge of the ways in which the job market and services operate. Empirically, language proficiency has for instance been shown to be strongly correlated with the likelihood of being employed (Dustmann, Fabbri, Preston, & Wadsworth, 2003) and, for a migrant, of having the practical information needed on arrival (Spencer et al., 2007).

Second, individuals are not bounded, isolated beings, but part of *families and social networks* (whether nearby or in country of origin) that may provide both constraints and opportunities across each integration domain. We could, in our model, situate these as part of an individual’s social capital, part of what they bring to the table, but this would be to individualise the processes at play. Whilst families and social networks of course overlap in most contexts, depicting them as having separate sets of effectors avoids the assumption that their effects will be homogenous, encouraging scrutiny of the multiple influences and diverse possible interactions in differing circumstances.

It is however not only individuals and families that are key to integration outcomes. Effectors relating to the *opportunity structures in society* are also known to be central to integration processes: openings in the labour market and housing markets, for instance, and barriers such as racism and discrimination. Opportunity structures cannot easily be separated from the fourth set of effectors, *policy intervention*: among local opportunity structures, for instance, government attitudes towards ethnic group initiatives have been found to impact on the level of involvement in local issues of minority organisations and their members in a range of European cities (Fennema & Tillie, 2004); but so have effectors related to the individual, including their gender and, in our transnational category of effectors, below, homeland politics (Garbaye, 2004; Kofman, Phizacklea, Raghuram, & Sales, 2000; Vermeulen, 2005).

A US study of the factors shaping the experiences of adult children of migrants found its results ‘almost frightening in revealing the power of structural factors’ on their lives, in which category they included the impact of family human capital, racism and a lack of intervention to lift the most disadvantaged out of poverty (Portes et al., 2005, p. 1032). In ‘new immigrant gateways’, lacking a prior history of in-migration, inexperience among service providers has been shown to affect the support migrants receive (Robinson & Reeve, 2006; Waters & Jiminez, 2005). A study of integration processes in European cities concluded that ‘the receiving society, its institutional structure and its reactions to newcomers are consequently far more decisive for the outcome of the process than the immigrants themselves’ (Penninx & Martiniello, 2004, p. 142).

Within the fourth set of effectors, *policy intervention,* the impact may be from mainstream policies or targeted at those who are marginalised, and, whether national or local, can facilitate or impede integration processes. Policy may be directed at opening up opportunities for employment, social interaction or civic participation, or to countering negative attitudes and unlawful discrimination. Alternatively, policy measures, including restricted entitlements attached to immigration status, may limit opportunities for migrants to access jobs, services or welfare support (Oliver, 2013). The stated rationale for a policy measure may be to facilitate integration but that rationale may be contested in civil society - as in the income threshold below which a migrant spouse may not be sponsored from abroad (MRN, 2014).

The final set of effectors is that of *transnational factors* that cut across our former categories; relating to the individual’s country of origin and the policies of that country. The ease and low cost of international travel and, in particular, of means of communication such as skype or viber, and the increasing interest of governments in ‘sending countries’ in their expatriate population (De Haas, 2006) mean that integration processes are less bounded by location and separation from past lives than for previous generations. The impact of transnational factors on integration processes, such as the significance of remittances, home-ownership in country of origin and or ‘home town associations’, is increasingly the focus of study, reflecting the ‘transnational turn’ in migration studies (Gidley & Caputo, 2013, p. 15), including those authors’ own analysis of the impact of transnational factors on residential integration. It is often assumed that retaining ties to country of origin militate against full participation in the new society but that assumption has been found to have little basis in empirical research. Rather, transnational connections and activity can coexist with participation across integration domains (Joppke & Morawska, 2003, p. 22; Snel et al., 2006).

### Implications of this approach for empirical application

Conceptualising integration in this way both facilitates design of effective research methodology and creates challenges for research in capturing the complexity of integration processes. Such an approach ensures that a study does not fall into the trap of assuming that focusing investigation on one domain of integration, the cultural perhaps, to the exclusion of other domains will provide comprehensive findings on ‘integration’. It requires clarification of the domains of integration to be covered and analysis of the implications of experiences in one domain for those in another. How has experience in the labour market impacted on social participation; or experience of civic participation impacted on identity at the local level?

Second, it ensures that a study does not focus exclusively on the characteristics that the individual brings to the table to the exclusion of the structural opportunities and barriers that they face – notwithstanding the methodological difficulties of so doing. It is easier to measure the education and skill level of a cohort of new migrants seeking employment, for instance, than to assess the relative job opportunities they faced in different parts of the country, at different times, relative to those skills. Conceptualising integration in this way nevertheless requires an attempt to identify the full range of effectors that may impact significantly (often in gendered ways) on integration processes, or at least to acknowledge those that have not been considered because of the methodological difficulty of so doing. Has limited choice of affordable housing impacted on levels of segregation? What has the impact been of policies in country of origin, on remittances, perhaps, or dual-citizenship? The goal is to go beyond merely noting the contexts in which integration processes are taking place; rather, it is to identify *within* those contexts which are the factors which have a significant impact on integration outcomes, and hence merit the greatest attention.

Third, the model requires us to take into account the temporal dimension: how the actors, processes and impact of effectors change over both chronological and life course time. It reminds us that integration ‘outcomes’ are merely a snapshot in an ever changing set of processes; not the end state of a linear, unidirectional, static process but an ebbing and flowing shaped by many factors, over many years, including life-stage events related or unrelated to the migration process. Finally, encompassing the impact of transnational contexts helps to overcome the methodological nationalism that can otherwise constrain our understanding of the processes at play (Wimmer & Glick Schiller, 2002).

### Case study: marriage migration

The topic of marriage-related migration brings into focus several dimensions of this conceptualisation of integration. Not only is the potential importance of *family* relationships reinforced, but the centrality of *gender* and of *life course* events become apparent.

*Family* as a concept operates on varying scales – the couple, nuclear family, broader extended family, or household - any of which may be relevant for processes of integration. Decisions on employment, for example, are often taken at a couple or household level, taking other labour and caring demands into consideration. In the case of transnational marriage, the integration of the migrant spouse is entangled with the lives of non-migrant family members. The family is thus not merely a context which may facilitate or impede the integration of an incoming spouse. The spouse is part of broader processes at the family level and its many impacts need to be taken into account.

We saw that integration is inherently temporal. Integration processes unfold over chronological (and perhaps generational) time, but they also vary over the *life course* (see for examples, McDonald & Elder, 2006; Morgan, Neal, & Carder, 1996; Wellman, Wong, Tindall, & Nazer, 1997, on fluctuations in individual’s social capital). Marriage is often a key life course event – what Johnson-Hanks has termed a ‘vital conjuncture’: ‘a socially structured zone of possibility that emerges around specific periods of transformation in a life or lives’ (Johnson-Hanks, 2002, p. 871). Hence, Rytter interprets Danish Pakistani’s choice of a ‘love’ marriage within Denmark (rather than an arranged transnational marriage) as an action of symbolic mobility which aligns them with the modern Danish identity favoured by the State, whilst Charsley points to the potential for marriages between British Pakistanis and spouses from Pakistan to increase the British partner’s transnational engagements and orientation (Charsley, 2013, p. 51–52). For the migrant spouse, the changes wrought by marriage are self-evident, but even where neither partner crosses a national border, marriage commonly entails geographical mobility of at least one spouse. Hence we must seek to tease out the differing impacts of the marriage from those of migration. In many South Asian cultures there is a strong convention that wives will move to husbands’ households (Bradby, 2000; Charsley, 2005), but the phenomenon of wives moving with or to join husbands has also been noted in the literature on Europe and North America. Both this common geographical mobility and the marriage itself have consequence for several of our domains (such as loss or alteration of social networks, changes of employment and housing).

In the South Asian groups which are the focus of our study, unmarried parenthood is rare, and childbearing usually follows quickly from marriage, often reinforcing gendered roles so that women, for example, may withdraw from full time paid employment. Given these gendered roles, life course instability of social capital may be particularly dramatic among women (McDonald & Elder, 2006). (Heterosexual) marriage, the family and life course are inherently gendered, but as Anthias and Pajnic (2014) argue, *gender* should be fundamental to understandings of integration. Not only are opportunity structures such as the labour market patterned by gender but women are key sites for the construction of community identities (with implications for the policing of their behaviour), immigration and integration policies have gendered impacts, and gendered constructions loom large in integration discourse.

Migrant men and women often face differing expectations, opportunities, constraints and vulnerabilities in integration processes. Migration for women may enhance autonomy and prestige but also social isolation (Decimo, 2005), and migrant wives’ dependency on husbands for their immigration status can be a barrier to their social and labour market participation (Özdemir et al., 2004). The vulnerability and dependency of migrant husbands are less commonly discussed but may combine with the transnational breadwinner’s ‘double burden’ of supporting families in both countries of settlement and origin to increase pressure on wage earning, leaving little room for developing social networks (Charsley, 2013; Charsley & Liversage, 2015). Both migrant men and women’s prospects for social and labour market integration may also be affected by gendered stereotyping and discrimination, emphasising the importance of an intersectional appreciation of the role of gender in integration processes. Once again we must not confuse impacts deriving from gender, regardless of migration experience, from those deriving from transnational marriage, albeit empirically no easy task.

In European research and policy discourse, the term integration is commonly also applied to European-born ethnic minority populations of migrant origin (Crul & Vermeulen, 2003), but this usage is controversial amongst British academics. Nevertheless, the constitutive processes of integration in our model – participation and processes of change in the various domains – are often used to measure ethnic inequality or cultural change among British ethnic minorities (cf. Modood et al., 1997), or indeed inclusion of other marginalised groups. Setting aside the question of the optimal term used for these processes, in our framework we are able to explore processes in the same domain in relation to both migrant spouses and their non-migrant ‘receiving’ families, exposing interactions and enabling comparison.

### Empirical application

Our project on marriage migration provided an opportunity to operationalize our model of integration in empirical research. We outline our methodology here to illustrate the way in which this can be done, but also constraints that can be faced in identifying the full range of effectors at play.

The project combined analysis of existing survey data with semi-structured interviews to explore, comparatively, the relationships between marriage migration and integration processes in two of the British ethnic groups with the highest levels of transnational marriage: Pakistani Muslims and Indian Sikhs^4^. The former are often problematized in integration discourses, sometimes with explicit reference to the frequency of ‘homeland’ marriages, whilst the latter has been considered a model minority in terms of integration (e.g., Goodhart, 2013). In developing a background statistical portrait of the correlations between transnational marriage and a range of ‘indicators’ of integration processes, we were constrained by the limited data sources that could offer appropriate representation of the relevant groups, and variables: the Labour Force Survey (LFS) and the ‘Born in Bradford’ dataset. From the LFS we were able to compare highest level of education attained and labour market status of couples where both partners were UK born or arrived under the age of 18, and those in which one spouse migrated to the UK as an adult. The ‘Born in Bradford’ dataset (a cohort study of 13,500+ babies born in Bradford in the years 2007–2011, and their parents) offered data on only one of our ethnic groups (Pakistanis) and in one location, but contains a wider range of relevant information. Using cross tabulations and multivariate regression we examined the relationship between country of birth, ethnicity and religion of spouses and a range of indicators such as labour market participation, occupation, income, political participation, experience of discrimination and subjective wellbeing. The limitation of these sources in the range of information they provided and their ability to provide insights into the processes underlying these ‘indicators’ reinforced the importance of the qualitative research.

For the qualitative interviews we adopted an innovative sibling-pair sample, comparing the experiences of siblings where one has married within the UK ethnic minority population and the other has married transnationally. The logic of this design was to address directly the implied or explicit suggestion that marriage within the UK ethnic population is preferable in integration terms to transnational marriage (Home Office, 2002; Kofman et al., 2015). The sibling pair design holds constant some variables which may be independently related to integration but may also influence the likelihood of transnational marriage and therefore give rise to the danger of inappropriate attribution of causality to marriage choices. Siblings are likely to share a number of relevant background characteristics (e.g., region of parental origin, parental socio-economic status, *zat*, faith). The inclusion of couples who had experienced the changes associated with marriage but without the involvement of international migration permitted reflection on the role of family related life course effectors. Interviewing (wherever possible) both spouses in a marriage and other family members (in the sibling sets) enhanced the potential to explore the intertwining of processes of integration within couples and families. Given the importance of gender to both marriage and integration processes, for the purposes of recruitment and comparative analysis the transnational couples were subdivided into ‘migrant wife’ and ‘migrant husband’ couple types^5^. The sample allowed for a range of lengths of time since marriage/migration to allow for the development of integration processes.

To address variation in local structural opportunities and constraints, important in our model of integration, the original survey design located the study in two contrasting cities, Bristol (in the South-West of England) and the conurbation of Bradford and Leeds (in the North). Those sites were selected to reflect differing patterns of residential concentration (cf. Ahmad, 2003), local economies, and region/social group of origin. The multiple sites were designed to permit exploration of local structural opportunities and barriers to integration processes, avoiding overemphasis on individual factors. Challenges with recruiting the sibling-pair sample^6^, however, meant that recruitment was expanded to include Birmingham and the Midlands, reducing our capacity for systematic comparison between sites (and hence, crucially, findings on the impact of place). Interview questions addressing the area of residence did provide some limited data.

Interviews schedules were structured to take the interviewees through their experiences in each integration domain, exploring potential effectors and the impact of experiences in one domain upon those in another. Thus questions relating to the structural domain, for instance, explored experiences *inter alia* in relation to the labour market, health care, and welfare benefits, seeking to identify the full range of potential effectors from education and skill levels through cultural expectations to non-recognition of qualifications and perceptions of discrimination. In the social domain the interview explored social networks and the frequency and places of contact, teasing out the effectors which shape those experiences including family relationships, cultural expectations and the opportunities provided by pre-marriage contacts for those who were UK born. Questions in the cultural domain explored issues from language and media usage to religious practices and, in the civic/political domain, of active engagement in organisations and formal engagement in the democratic process. In the identity domain feelings of belonging in relation to neighbourhood, city and nation were explored, again teasing out the factors that may contribute to this. To reflect the temporal nature of integration and the importance of the life course and life course events (particularly marriage), the interviews took a life history format, with particular attention to changes associated with or occurring after marriage/migration.

The innovation in this approach did not lie in the issues covered by the interview questions, all resonant of earlier studies on aspects of integration, but in the deliberate structuring of the interviews to cover each integration domain; in the focus on the effectors that explain experiences; and in the exploration of the impact of experiences in one domain on another. Coding of interviews using NVivo enabled these connections to be identified, and comparison to be made between couple types within and between ethnic groups.

Whilst, as noted above, parts of this complex research design proved challenging to implement, the data generated nevertheless provides rich material with which to explore and compare the relationships between marriage migration and processes of integration, revealing patterns and variation within and between groups which we will set out more fully elsewhere. Here, three examples of couples from our Pakistani sample serve to illustrate the way in which our model can illuminate the multiple processes at work.

**Case A** illustrates a key but under explored feature of integration processes: that integration in one domain can proceed to a far greater extent than in another. It also illustrates the entanglement of integration processes within family relationships, and the key role played by life course events: in this case marriage and having children. Case A is a British Pakistani woman whose family withdrew her from school at the age of 12 to look after her ill mother. She had an early transnational marriage and has never been in paid employment. In many ways, then, her early life reflected stereotypes of ‘unintegrated’ Muslim women. Her husband is a semi-skilled manual worker, with limited social networks. Making do on his income alone, the couple and their children live rent-free in a house owned by her brother. Her marriage, however, has released her from her gendered responsibility to care for her mother and once the couple had children she began to extend her social networks across ethnic groups through participation at parents’ activities at the school and is now an active contributor to its fundraising activities. Whilst she remains inactive in the labour market, she has actively developed wider participation in the social and civic domains. One consequence of her transnational marriage is that her in-laws are in Pakistan. This, combined with her migrant husband’s dependence on his wife’s family (for accommodation), reduce the potential role of affinal (in-law) family responsibilities and their influence as effectors on her opportunities, in contrast to the conventional Pakistani expectations of the role of a daughter-in-law. Indeed, we find some evidence among our British Pakistani sample in support of the Lievens’ hypothesis – that women may seek transnational marriage for ‘modern’ reasons such as avoiding in-control (Lievens, 1999).

**Case B** illustrates a further feature of integration processes that is insufficiently understood: the impact of participation in one domain on another. It also demonstrates the range of effectors that can contribute across domains. Case B concerns a Pakistani husband, now divorced, who worked in a warehouse thanks to an introduction made by his wife’s relatives. He enrolled on an English course but was so tired from his long working hours that the teacher said there was no point in his continuing to attend. He changed jobs to work in the building trade, learnt his trade from fellow Pakistanis but also from white British workmates, and improved his English through conversation in that environment. He then felt able to enrol on a series of college courses and now has his own building company. In this case, the migrant spouse benefits from his ‘receiving’ family’s social capital in obtaining employment but the low wages typical of both British Pakistani and migrant employment opportunities, combined with the ‘double responsibility’ (Charsley, 2005) of contributing to both the UK household and remitting to family in Pakistan (a transnational effector), have impacts for both his time and linguistic skills which limit his participation in the social domain. His second line of employment, however, offered contrasting opportunities for employment, economic gain, and social engagement.

**Case C** illustrates the way in which a policy effector can operate as a barrier to integration despite apparent advantages in terms of effectors relating to individual human capital, and to the family. It also speaks once more to the importance of life course issues. Case C is a Pakistani migrant wife who has an MA in English Literature and who has taught at a university in Pakistan. She needs to convert her qualifications in order to teach in the UK and both she and her family are keen that she should do so. As a recent migrant, however, she cannot apply for a student loan and the current fee structure puts this training beyond the family’s economic reach. By the time she gains access to student funding, it is likely that she will have entered motherhood, when caring responsibilities may well provide a new barrier. For other Pakistani migrant and non-migrant women in our sample who aspired to paid employment, their engagement in the labour market relied heavily on their family’s assistance with childcare (for migrant women this meant their in-laws whilst for non-migrants natal families also sometimes provided this support, increasing the range of possibilities). Alternatively, they may wait until their children are older – one Pakistani migrant wife becoming a successful entrepreneur in an ethnic niche industry once her children were in their teens.

## Conclusion

We have argued that enquiry into the factors which impact on ‘integration’ processes requires clarity on the nature of the integration processes in which individuals are engaged across related domains, the intersection of those processes and the factors that may affect their operation over time. Only in this way, in our case study on the relationship between marriage migration and integration, could we identify the potential relevance of marriage with a partner from abroad among the full range of factors at play. Acknowledging that the term ‘integration’ is suboptimal, we presented a heuristic model of integration processes capable of empirical application. It is characterised by identifying the domains in which integration processes take place (structural, social, cultural, civic-political and identificational), their multi-directional, spatial and temporal character, and their interdependence: experience in one domain impacting on experience in another. The outcome may be greater participation in one domain than in another; and participation in (or identification with) a sub-section of society in that or all respects.

Our model highlights the importance of the ‘effectors’ that facilitate or impede integration processes, categorising them as related to individuals; families and social networks; society (the opportunity structures that it offers); policy intervention and transnational. Identifying families here as one prime source of effectors helps to focus the spotlight on life course events such as marriage and having children: events which may impact significantly on integration processes regardless of the transnational experience of the parent. It also serves to remind us of the centrality of gender, across domains.

On our choice of case study, we argued that spousal migration is important for the study of integration not just as a significant source of cross-border mobility but because it highlights several often neglected aspects of integration, not least the family, life course and gender, with relevance beyond this particular migration stream. Whether moving through ‘family’ migration channels, or as labour migrants or refugees accompanied by or leaving behind dependents, family relationships are key to migration motivations and experiences, and therefore to processes of integration. Perhaps, as Cooke has recently suggested, in some sense ‘nearly all migration could be defined as family migration’ (Cooke, 2008, p. 260).

Drawing on our study on marriage migration we demonstrated the value of our model, ensuring as it does that a study does not fall into the trap of investigating experiences in one domain of integration without regard to the impact in others, or fail to recognise the full range of effectors which may be at play. It requires that we focus not only on the characteristics that the married couple bring to the table but on the structural opportunities or barriers they may face; and recognise that the processes of integration are continually ebbing and flowing: outcomes are merely a snapshot at one point in time.

Outlining the mixed method deployed for the study, we showed that a background statistical portrait could only be developed across some indicators of integration, while qualitative interviews enabled us to explore experiences across integration domains – analysing their inter-relationship using the coding facility provided by NVivo. We shall report on the findings elsewhere but used three cases to illustrate how the qualitative material can illustrate the multiple, nonlinear, integration processes at work.

The complexity of integration processes demand complex research designs which may be challenging to implement. Operationalising our concept of integration did indeed present a series of challenges: the range of potential effectors to consider within each domain of integration, and difficulty of identifying and measuring their relative impact. Processes in some domains are easier to measure than others – mobility in the labour market, for instance, identified more easily than shifting attitudes of either the migrants or existing residents. It is easier to identify micro factors of which individual interviewees are aware than structural barriers such as lack of opportunities in the labour and housing markets which can differ between regions and neighbourhoods and change over time. Lines of causality may not always be clear, nor the extent to which the impact of a life changing event such as marriage would have occurred without the added dimension of a partner from abroad.

These challenges mean that there will be parts of the picture that emerge more clearly than others. We anticipate that our findings may provide more data on the ways in which human capital, marriage and family impacted on integration processes across domains than on key societal and policy effectors. What our model does enable us to do, however, is to situate our analysis within a broader understanding of the range of potential effectors. In that way we can avoid falling into the trap of assuming that the effectors closest to the individuals and to the newcomers in particular are telling the whole story of the forces at play.

## Footnotes

1 The research involved new analysis of the Labour Force Survey household files, semi-structured interviews with 35 Indian Sikh (18 men, 17 women) and 43 Pakistani Muslim (17 men, 26 women) participants, and supplementary focus groups involving 25 further participants.

2 COUNCIL DIRECTIVE 2003/86/EC of 22 September 2003 on the right to family reunification: http://eur-lex.europa.eu/legal-content/EN/TXT/PDF/?uri=CELEX:32003L0086&from=EN (para 4).

3
http://dictionary.cambridge.org/dictionary/british/integrate?q=integration

4 Employing these categories allows us to engage directly with policy and academic discourse in the field of marriage migration and integration, but we do not assume identification with these groups, nor suggest that interesting insights could not be gained from exploring processes affecting other populations. American and Thai spouses settling in the UK, for example, are an interesting pair as both flows of marriage migrants are highly feminised (Charsley, Van Hear, Benson, & Storer-Church, 2012) but only Thai marriage migration tends to be presented as problematic. Neither group, however, are key to contemporary discourses surrounding marriage migration and integration, and moreover, do not occur in large enough numbers to allow quantitative analysis of the kind we have undertaken in this project (see full report at: http://www.bristol.ac.uk/ethnicity/projects/mmi/).

5 This distinction was also made in the quantitative analysis.

6 Which also meant that whilst sibling pair ‘sets’ remained the core and majority of our sample, ‘extra’ interviews were conducted to provide additional data related to categories of participant for which recruitment proved challenging.
